# Supplementary material for: Inactivation of SARS-CoV-2 and influenza A virus by dry fogging hypochlorous acid solution and hydrogen peroxide solution
Source: PLoS One. 2022 Apr 7;17(4):e0261802. doi: 10.1371/journal.pone.0261802 (PMC8989197; doi:10.1371/journal.pone.0261802)
Supplement: Attachment — Submitted filename: PONE-D-21-38754R1_Response to reviewers.docx [file pone.0261802.s003.docx]

Thank you for the opportunity to revise our manuscript, **PONE-D-21-38754R1** entitled “**Inactivation of SARS-CoV-2 and influenza A virus by dry fogging hypochlorous acid solution and hydrogen peroxide solution**” by Urushidani, M. et al. We appreciate the detailed review and suggestions made by the reviewer #1. We herein present the Reviewers’ comments in bold, followed by our point-by-point responses. Changes made in the revised manuscript are marked using track changes.

**Reviewer #1: I think you still need to make the figure captions more concise. A lot of the verbiage in the captions I believe is already described in the materials/methods section, or described in the Results section.**

Thank you for your suggestion. We shortened the figure legends.

**I think the Discussion section could be made more concise as well, especially the last two paragraphs. this seems like a repeat of the Introduction.**

Thank you for your suggestion. We removed some sentences that overlapped with the Introduction section, and made the Discussion section more concise. Please let us remain some sentences “overlapped” with the Results section, because we would like to emphasize the results. We hope the reviewer agrees with our opinion.

**In the abstract and elsewhere, where you say that it is also transmitted by contact infection, i think maybe you should say it may be transmitted by surface contact. Use the word "may".**

Thank you for the suggestion. We added “may” in the corresponding sentence in the Abstract section.
